# Instituting coronavirus disease 2019 testing: opportunities and challenges of molecular laboratory diagnosis in a Southern Nigerian teaching hospital

**DOI:** 10.1093/inthealth/ihae023

**Published:** 2024-03-13

**Authors:** Iriagbonse I Osaigbovo, Isaac O Igbarumah, Darlington E Obaseki

**Affiliations:** Departme nt of Medical Microbiology, School of Medicine, College of Medical Sciences, University of Benin, Benin City 300213, Edo state, Nigeria; Department of Medical Microbiology, University of Benin Teaching Hospital, Benin City 300001, Edo state, Nigeria; Molecular Diagnostic and Virology Laboratory, University of Benin Teaching Hospital, Benin City 300001, Edo state, Nigeria; Office of the Chief Medical Director, University of Benin Teaching Hospital, Benin City 300001, Edo state, Nigeria

**Keywords:** COVID-19, global health, laboratory preparedness, Nigeria, outbreak, PCR

## Abstract

The coronavirus disease 2019 pandemic emphasised the importance of laboratory preparedness, including molecular diagnostic capacity, in the control of infectious disease outbreaks. This article reflects on diagnostic capacity-building opportunities presented by the pandemic, the challenges experienced along the way and the lessons learned from the perspective of a university teaching hospital in Southern Nigeria. We advocate for these lessons to inform strategic planning for laboratory preparedness at subnational, national and continental levels.

## Introduction

The coronavirus disease 2019 (COVID-19) pandemic began in China in December 2019, constituting a public health emergency of international concern (PHEIC) from 21 January 2020 to 5 May 2023 and causing multi-sectoral disruptions across the globe. Against the backdrop of a WHO directive to ‘test, test, test’, >700 million cases of COVID-19 were detected globally, emphasising the importance of diagnostic capacity in responding to infectious disease outbreaks.^1,2^ Highly sensitive and specific nucleic acid amplification tests, typified by real-time reverse transcriptase polymerase chain reaction (RT-PCR) and popularly dubbed molecular diagnostics, were instrumental to the outbreak response, enabling early detection, expedient clinical management and objective assessment of outbreak containment, not to mention facilitating the development of drugs and vaccines.

The capital investments required to deploy molecular testing posed a significant hurdle for many African countries to scale. This was partially overcome by the emergence of rapid antigen detection methods. However, the subpar sensitivity of these cheaper alternatives implied that they remained complementary tools to the gold standard molecular test.

The current article summarises the outbreak response of an academic tertiary hospital in Southern Nigeria from a diagnostic perspective. Importantly, we reflect on the opportunities presented by the pandemic to build molecular testing capacity, the challenges encountered and the lessons learned from the experience.

## Setting

The University of Benin Teaching Hospital (UBTH) is located in Benin City, Edo state in Southern Nigeria. This acute care tertiary facility catered for the most severe cases of COVID-19 during the pandemic, which commenced in the state on 23 March 2020.^3^ The hospital's response, which has been previously described, adopted a multi-pillared approach, including a dedicated diagnostic pillar.^3^ The mandate of this pillar, which was to oversee laboratory testing for severe acute respiratory coronavirus 2 (SARS-CoV-2), the aetiological agent of COVID-19, initially revolved around specimen collection and referral, because the facility lacked compatible molecular testing equipment. However, the hospital leadership, in anticipation of a surge in COVID-19 cases, instituted testing onsite by entering collaborative agreements to access appropriate equipment and reconfiguring the existing laboratory's layout for enhanced biocontainment.^4^

## Activities of the diagnostic pillar: the rise and fall of COVID-19 testing

**Figure 1.**
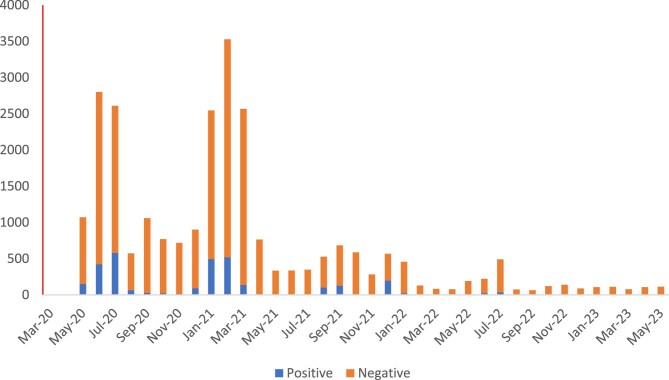
COVID-19 testing volumes in the UBTH molecular virology laboratory (May 2020 to May 2023). Peaks in sample volumes were experienced in June 2020, February 2021, September 2021, December 2021 and July 2022. These peaks roughly coincide with distinct pandemic waves. The declining number of tests in successive peaks depict waning public health interest in laboratory testing as the pandemic progressed.

The molecular virology laboratory was activated for COVID-19 testing on 10 May 2020, quickly becoming the diagnostic and public health surveillance hub of the state.^4^ By the end of the PHEIC on 5 May 2023, a total of 26 694 samples had been processed, of which 3535 were positive. The distribution over the 3-y period is shown in Figure 1. Promptly reporting all test results to relevant stakeholders constituted another crucial task of the laboratory. The diagnostic pillar also evaluated four rapid antibody diagnostic kits and six antigen tests, using RT-PCR as the reference test, during the pandemic. Specimens received for molecular testing were submitted for next generation sequencing contributing to genomic surveillance in the state. Finally, the pillar was responsible for providing evidence-based guidance by periodically appraising the scientific literature to stay abreast of developments in the field of SARS-CoV-2 testing.

## Opportunities

Instituting onsite testing boosted the hospital's overall pandemic response, positively impacting patient management, infection prevention and control and health-worker safety. In addition, molecular testing provided other unique opportunities. In terms of personnel development, the laboratory staff benefitted from sponsored technical, safety and data-management training, resulting in new skills and competencies. Second, the presence of high-volume molecular testing attracted research and other collaborators to the hospital. For example, the UBTH was a co-awardee of a grant to implement a multi-centred diagnostic evaluation of rapid antigen test kits in Nigeria. It was also selected to execute a funded health worker infection case-control study and as a sentinel site for acute febrile illness surveillance in the country.

## Challenges

Setting up and sustaining COVID-19 testing presented obstacles that the UBTH management tackled with varying degrees of success. A lack of suitable testing platforms was the primordial challenge that needed surmounting and this was achieved by collaborating with a research centre in the University of Benin, which provided an open PCR machine. Another formidable challenge was the manpower required to cater to surges in testing. A general reduction in non-COVID-19 care at the start of the pandemic meant that staff from the general medical microbiology laboratory could be conveniently redeployed to circumvent this challenge. However, these staff had no previous experience with molecular testing and required considerable training before they could offset the workload. With the lifting of lockdown measures and rebound in services across other parts of the hospital, some of these personnel resumed regular duties, leaving the remaining staff in the molecular laboratory overworked, especially during the peak of subsequent waves.

High sample volumes and a limited workforce had a negative impact on diagnostic turnaround times (DTAT). This was especially problematic for triaging cases in the emergency area. The laboratory responded by dual-tracking the testing process, with priority given to clinical samples. Eventually, with the procurement of additional testing platforms, there was a marked improvement in DTAT for all specimens.

Funding was, and remains, perhaps, the foremost challenge for sustaining molecular testing. Molecular testing for COVID-19, although capital intensive, is offered for free to all citizens due to the disease's public health importance. Although reagents and consumables are provided by the Nigeria Centre for Disease Control (NCDC), the laboratory and hospital management bear the brunt of power, connectivity, storage, administrative and maintenance costs, among others.

## Lessons learned

Instituting COVID-19 testing, although challenging, provided valuable insights, as enumerated below:

1. *Invest in open PCR systems*: Although in existence since 2011, the UBTH molecular virology laboratory possessed only a proprietary, or closed, PCR machine used specifically for HIV testing.^4^ This machine was not compatible with any available SARS-CoV-2 assays. Acquiring an open PCR machine enabled us to initiate and maintain testing with a variety of SARS-CoV-2 assays. This proved useful for coping with supply chain disruptions occasioned by the pandemic. It also broadened the scope of pathogens that can be tested for in the future.
2. *Keep both human capacity and infrastructure for molecular diagnosis patent*: The existing human capacity for performing HIV PCR in the facility made initiating COVID-19 testing in UBTH relatively easy, despite the small number of skilled personnel.^4^ However, the lack of competent hands resulted in valuable manpower hours expended on training in the heat of the pandemic. Thus, a critical mass of laboratory technologists needs to undergo training in molecular techniques to enable seamless task-shifting during outbreaks. To achieve this cost-effectively, molecular testing facilities should remain active, obviating the need for technologists to travel far from their bases to acquire training. Thus, it is essential that molecular laboratories activated in Nigeria during the COVID-19 pandemic be kept patent by engaging them in testing for other diseases of public health significance (e.g. Lassa fever, mpox, cholera and neglected tropical diseases), following extensive diagnostic network optimisation by the NCDC. This should be a joint responsibility of the respective institutions and the government.
3. *Build the research capacity of laboratory personnel*: Research is an important aspect of overall outbreak response and is crucial for developing and evaluating effective diagnostics. Deficiencies in research capacity, which were mostly quantitative (the few laboratory staff with research experience were constrained by involvement in operations), but also qualitative (e.g. a lack of grantsmanship abilities), meant that opportunities to evaluate diagnostic methods and strategies, such as pooled sample testing, remained unexplored. Building grantsmanship and research capacity in the laboratory may facilitate leveraging on the other lessons learned. For instance, grant funding could be used to procure equipment and execute testing for infectious diseases of public health relevance. Training and capacity building can also be built into grant applications. This will be a way of sustaining and enhancing already acquired competence, as well as diversifying it to include advanced capabilities such as bioinformatics and genomic sequencing.

The aforementioned lessons have been instructive in the UBTH's strategic investments in molecular laboratory equipment, personnel and research capacity. For instance, the management judiciously channelled COVID-19 intervention funds provided to all tertiary hospitals by the federal government into the purchase of MIC and QuantiStudio real-time PCR systems. We are also actively interfacing with the NCDC to include our laboratory in testing for other diseases of public health significance, notably Lassa fever. Finally, a renewed hospital-wide drive for grantsmanship is expected to specifically target the molecular laboratory.

## Conclusion

Laboratory preparedness, a critical aspect of pandemic response, has been deeply impressed on governments, public health institutions, health facilities and indeed all stakeholders during the COVID-19 pandemic. The pandemic presented not only a challenge, but an opportunity to build on the existing capacity for molecular diagnosis in the UBTH. Although COVID-19 is no longer a public health emergency, molecular testing capacity for infectious diseases remains a priority at national and subnational levels to ensure laboratory preparedness for, and response to, future pandemics. The lessons learned from instituting COVID-19 testing at the UBTH molecular virology laboratory are being leveraged to inform a strategic plan focused on investing in personnel, equipment, research and development. Similar responses are required at national and continental levels.

## Data Availability

Not applicable as no data were specifically generated for the purpose of this paper
